# Cardiometabolic multimorbidity and cognitive-functional transitions across international ageing cohorts

**DOI:** 10.3389/fpubh.2026.1920385

**Published:** 2026-09-10

**Authors:** Ming Li, Xiaoyu Pan, Xi Li, Xian Wu, Jiale Cai, Lei Li, Hailong Lu

**Affiliations:** 1 Department of Geriatrics, The Affiliated Hospital of Xuzhou Medical University, Xuzhou, Jiangsu, China; 2 National Demonstration Center for Experimental Basic Medical Science Education, Xuzhou Medical University, Xuzhou, Jiangsu, China

**Keywords:** cardiometabolic disease, cognitive ageing, functional limitation, international cohorts, longitudinal analysis, multimorbidity

## Abstract

**Background:**

Cardiometabolic multimorbidity (CMM) may accompany changes in cognition and everyday function in later life. We reassessed these associations across international ageing cohorts while addressing outcome structure, unequal threshold severity, measurement precision, repeated observations, mortality and non-death attrition.

**Methods:**

We harmonised repeated observations from HRS, ELSA, CHARLS, KLoSA, MHAS and SHARE. Within-interval change in a complete four-condition CMM count (hypertension, diabetes, heart disease and stroke) was used in continuous analyses, whereas transition analyses used interval-baseline CMM4. Fixed-reference, non-overlapping cognitive and functional composites were constructed by cohort. Continuous outcomes were paired cognitive and functional worsening, analysed with participant-clustered Gaussian GEE and a CMM-by-domain interaction. Binary outcomes were any cognitive and any functional transition using cohort-wave-specific thresholds targeting 20% occupancy; sensitivity analyses included 15, 25%, historical −0.43/+0.43, stricter −0.67/+0.67 and persistent-state definitions. Logistic GEE estimates were pooled using REML with Hartung-Knapp confidence intervals.

**Results:**

The data included 236,088 people, 172,518 with at least two waves, and 523,570 scheduled-wave or terminal-death intervals. In 361,562 paired intervals across six cohorts, each additional CMM condition accumulated was associated with cognitive worsening (*β* = 0.0459, 95% CI 0.0243 to 0.0675; *I*^2^ = 74.3%) and functional worsening (*β* = 0.0960, 0.0809 to 0.1111; *I*^2^ = 41.8%). The functional-minus-cognitive contrast was 0.0534 (0.0325 to 0.0742; *I*^2^ = 51.6%). Among 266,127 intervals beginning in the distribution-defined unimpaired state, each additional baseline CMM condition was associated with any functional transition (OR = 1.33, 95% CI 1.27 to 1.40; *I*^2^ = 64.9%) but not with a common any-cognitive transition effect (OR = 1.01, 0.95 to 1.07; *I*^2^ = 81.8%). The functional-to-cognitive ratio of odds ratios was 1.32 (1.23 to 1.42). Persistent any-functional transition was associated with CMM (OR = 1.39, 1.33 to 1.45; *I*^2^ = 0%), whereas persistent any-cognitive transition did not (OR = 0.99, 0.89 to 1.10; *I*^2^ = 77.0%). Fixed reference-wave thresholds, disease-restricted exposures, cohort exclusions, KLoSA ADL-only scoring and inverse-probability weighting preserved the functional contrast.

**Conclusion:**

CMM was associated with worsening in both continuous domains, with a larger functional coefficient, and with more consistent distribution-defined functional than cognitive transitions. Associations were mainly synchronous, although a smaller delayed cognitive association was observed. Binary states are not diagnoses of mild cognitive impairment, dementia or clinical dependence; findings do not establish causality or a validated screening rule.

## Introduction

Multimorbidity is common in later life, when chronic disease accumulation coincides with changes in cognition, physical capacity and independence (1–3). Ageing and dementia-prevention frameworks therefore emphasise function, intrinsic capacity and modifiable cardiometabolic risk alongside disease counts (1, 4, 5). Cardiometabolic conditions often cluster and may identify people whose health is changing across several domains (4–8).

Most previous studies have examined cognition or disability separately (7–12). This is analytically convenient but does not fully reflect comprehensive geriatric assessment, in which cognitive and functional changes often co-occur and are considered alongside intrinsic capacity (13). Zhang et al. (14) modelled long-term joint trajectories of physical disability, depressive symptoms and cognition in a multicohort design. The present study instead examines within-person, adjacent-wave change in CMM and paired cognitive-functional change, together with transitions among explicitly distribution-defined cognitive-functional states.

Four-state descriptions can distinguish an isolated low cognitive score from an isolated high functional-limitation score and from joint impairment. However, a cognitive-only state is not equivalent to all cognitive impairment: anyone meeting both thresholds is assigned to the joint state. Domain-level conclusions therefore require outcomes that recombine cognitive-only with joint impairment and functional-only with joint impairment. Cross-cohort comparisons add further challenges because cognitive batteries differ, functional scales are floor-heavy, identical *z* cut-points can select unequal proportions, and wave-specific standardisation measures relative rather than absolute position.

We harmonised the cohort-level source files to address these issues. Our primary questions were whether CMM accumulation was associated with continuous worsening in cognition and function, whether the two coefficients differed in a formal paired-domain model, and whether baseline CMM was associated with inclusive any-cognitive and any-functional transitions under prevalence-matched state definitions. Isolated states were treated as secondary decompositions, and alternative thresholds, persistent states, mortality, attrition, standardisation and measurement reliability were evaluated explicitly.

## Materials and methods

### Study design and data sources

This observational longitudinal study used the Health and Retirement Study (HRS), English Longitudinal Study of Ageing (ELSA), China Health and Retirement Longitudinal Study (CHARLS), Korean Longitudinal Study of Ageing (KLoSA), Mexican Health and Aging Study (MHAS), and Survey of Health, Ageing and Retirement in Europe (SHARE) (15–20). The analysed releases were Harmonized CHARLS Version D with the locally processed 2020 wave; Harmonized ELSA Version G.3; Harmonized HRS Version D with RAND HRS 2020 Version 2; Harmonized KLoSA Version E.2 (2006–2020; DOI 10.34729/815E-WN35); Harmonized MHAS Version C.2; and Harmonized SHARE Version F.2 based on SHARE Release 9.0.0. KLoSA cognition was analysed in waves 3–7 after reconstructing its K-MMSE components from the wave files; SHARE cognition used waves 4–8. Other included waves are detailed in Supplementary Appendix 1 and Supplementary Table S1.

Participant-wave records were linked by cohort-specific identifiers. Exact interview year and month were converted to decimal years. Scheduled adjacent waves were defined from the cohort wave sequence, rather than simply pairing the next observed record. Intervals required a positive elapsed time. Participants could contribute more than one interval, and all primary models clustered covariance by participant. A sensitivity analysis excluded intervals shorter than 0.5 years or longer than 5 years.

### Cardiometabolic multimorbidity

The primary CMM4 count comprised hypertension, diabetes, heart disease and stroke. Each condition was harmonised as present or absent using declared valid and missing codes. For doctor-diagnosed chronic conditions, the first positive report was carried forward so that a later observed zero could not make an established disease disappear. A CMM4 value required all four components; interval change required the same four-component denominator at both endpoints. This eliminates changes caused solely by a changing component denominator.

A complete CMM5 sensitivity added high cholesterol in CHARLS, ELSA, HRS and SHARE when all five components were observed at both endpoints. A sustained-diagnosis sensitivity required a newly reported diagnosis to remain present at the next scheduled living interview; a terminal death record could not serve as diagnostic confirmation. To assess reverse diagnostic ascertainment and the contribution of stroke, we also analysed a two-condition diabetes-plus-stroke count and a three-condition CMM count excluding stroke. The primary transition exposure was baseline CMM4; the continuous exposure was unannualised within-interval change in CMM4.

### Cognitive and functional composites

Each cohort used one validated total or a fixed set of non-overlapping component measures, never a total together with its constituent items. Cognition was oriented so that higher scores indicated better performance; function was oriented so that higher scores indicated more limitation. Components were standardised using the first eligible common reference wave within each cohort and combined only when all declared components were observed. The resulting composite was then scaled to the reference-wave mean and standard deviation. Wave-relative standardisation was evaluated separately.

The cognitive inputs were: total recall, orientation, serial subtraction and figure drawing in CHARLS; total recall and orientation in ELSA; the validated 27-point total in HRS; the reconstructed 30-point K-MMSE from 15 non-overlapping scored items in KLoSA; immediate and delayed recall combined as a memory domain plus visual scanning in MHAS; and immediate and delayed recall combined as a memory domain, serial subtraction and verbal fluency in SHARE. Functional composites combined fixed ADL and IADL domains: 11 items in CHARLS, 13 in ELSA, 11 in HRS, 17 help-dependence items in KLoSA, 10 in MHAS and 12 in SHARE. Exact variables, ranges, exclusions and reference waves are provided in Supplementary Table S1.

Complete-case alpha and Pearson one-factor omega were calculated where at least three scored components were available. For two-component constructs, we report the inter-component Pearson correlation and Spearman-Brown coefficient rather than an underidentified omega. At HRS reference wave 5, the three non-overlapping component scores contributing to the validated 27-point total (tr20, bwc20 and ser7) summed exactly to cog27 in all 17,516 complete records; descriptive component-score alpha and one-factor omega were therefore calculated. KLoSA item-level reliability was audited separately after reconstruction.

### Outcomes and state definitions

Continuous outcomes were cognitive worsening and functional worsening, defined so that a positive value indicated deterioration. We used unannualised change and adjusted for exact interval length to avoid placing the same elapsed-time denominator in both exposure and outcome. The primary formal domain analysis stacked the two outcomes from intervals with both domains observed and estimated cognitive, functional and functional-minus-cognitive CMM slopes.

Binary cognitive-functional states were defined among living records with both domains observed. The primary thresholds were chosen separately within each cohort-wave on the common two-domain sample to target 20% in the low cognitive tail and 20% in the high functional tail. Because functional scores were discrete and floor-heavy, tied values were never split; the nearest observed threshold was selected, with the more stringent/lower-occupancy threshold chosen when deviations tied. The four states were unimpaired, isolated cognitive-only, functional-only and joint. Inclusive outcomes were any cognitive impairment (cognitive-only plus joint) and any functional impairment (functional-only plus joint).

Sensitivity definitions targeted 15 and 25% occupancy. A fixed-threshold sensitivity estimated the 20% cognitive and functional cut-points once at each cohort’s reference wave and applied those unchanged cut-points to all later waves, thereby preserving absolute position relative to the reference distribution. The historical definition used cognition *z* ≤ −0.43 and function *z* ≥ + 0.43, and a stricter definition used −0.67/+0.67. Under a standard normal distribution, ±0.43 corresponds arithmetically to approximately 33.4% in each tail. The historical definition was retained only as a reproducibility analysis and was not interpreted as a clinical or prespecified diagnostic threshold; its documentary basis and empirical consequences are detailed in Supplementary Appendix 1. Persistent endpoints required the same inclusive impairment at the next two scheduled waves. All threshold audits report achieved occupancy, ties, empirical percentile bounds, skewness, floor and ceiling occupancy.

### Covariates

Primary models included age, sex, education, exact interval length, baseline cognition and baseline function. Continuous change-on-change models additionally adjusted for baseline CMM4; in transition models, baseline CMM4 was the exposure. The principal adjustment additionally included depressive symptoms and psychiatric disease where available; the exact available terms were recorded by cohort. Sequential models then added smoking, BMI, physical activity, alcohol use, socioeconomic position and marital status where harmonisation permitted. Medication adherence was not available comparably. Polypharmacy could not be harmonised across enough cohorts and is reported as unavailable rather than silently omitted.

### Statistical analysis

Cohort-specific continuous models used Gaussian identity-link GEE. Binary outcomes used logistic GEE; primary binary estimates used the Mancl-DeRouen bias-reduced sandwich covariance. All GEE models used participant clusters and an independence working correlation structure (21, 22). Continuous stacked models allowed domain-specific slopes for CMM, baseline cognition, baseline function, interval length and covariates; cognitive was the reference domain. Binary stacked GEE formally estimated the functional-to-cognitive ratio of odds ratios. Transition models were restricted to intervals beginning in the distribution-defined unimpaired state.

Death was appended as a terminal absorbing state in CHARLS, HRS, KLoSA, MHAS and SHARE using verified death year/month fields; ELSA mortality follow-up was incomplete after 2012 and was not treated as a valid death endpoint. Cause-agnostic death was analysed as a competing destination in binary transition models and in a secondary participant-clustered multinomial discrete-time model fitted by Newton–Raphson optimisation (maximum 200 iterations; convergence required a successful optimiser flag and finite coefficients with positive standard errors). Non-death response was modelled from prior response history, CMM, cognition, function, mental health and sociodemographic variables. Stabilised inverse-probability-of-censoring weights were truncated at the first and 99th percentiles; deaths were excluded from the censoring model rather than treated as non-response (23).

Cohort-specific estimates were combined by random-effects meta-analysis with REML variance estimation and Hartung-Knapp confidence intervals (24, 25). Heterogeneity was summarised using *i*^2^ (26), and prediction intervals were reported when at least three cohorts contributed. Non-finite or non-converged estimates were excluded from pooling and recorded in a model-completion matrix. Held-constant-cohort analyses retained the same cohort set, while model-specific complete-case samples were reported explicitly. Measurement sensitivities excluded ELSA, excluded KLoSA, and replaced KLoSA’s 17-item help-dependence score with its seven-item ADL score.

To quantify sensitivity to unequal measurement precision, cohort-specific continuous coefficients and standard errors were divided by the square root of the corresponding reference-wave reliability estimate and then recombined with the same REML/Hartung-Knapp procedure. We also conducted a reviewer-requested latent-normal simulation calibrated to each cohort’s empirical CMM4-change distribution and baseline-to-end cognitive correlation. In 1,000 repetitions per cohort (maximum 30,000 intervals per repetition), independent classical measurement error was added at the observed reliability, the 20% cognitive threshold was re-applied without splitting tied values, and the resulting thresholded association was compared with its latent-score analogue. This simulation illustrates attenuation of a thresholded version of the continuous CMM-accumulation estimand; it is not a direct correction of the primary baseline-CMM4 transition OR (Supplementary Table S19; Supplementary Figure S2).

The temporal-boundary analysis was conducted at the participant level among people with at least four observed waves. Early CMM4 accumulation was related to later cognitive and functional change after adjustment for CMM4 and both domain scores at the boundary, age, sex, education, and the durations of the early and later periods. This analysis separates time periods but remains observational and does not establish direction of causation.

We evaluated discrimination using five-fold participant-grouped, outcome-stratified cross-validation. The base prediction model contained age, sex, education and baseline function; the expanded model also included baseline cognition. Each was fitted with and without CMM4. AUC and Brier-score uncertainty used 200 participant bootstrap samples. Standardised absolute risks compared CMM4 ≥ 3 with CMM4 = 0 among observed living endpoints. These are risk contrasts, not screening-test performance or numbers-needed-to-screen. Reporting followed STROBE guidance (27).

All analyses used two-sided tests. Harmonisation and modelling used Python 3.14.2 with pandas 2.3.3, NumPy 2.4.3, SciPy 1.17.1, statsmodels 0.14.6, patsy 1.0.2 and scikit-learn 1.8.0; KLoSA extraction used R 4.5.2 with haven 2.5.5. No participant-level data are included in the reproducibility package.

## Results

### Cohort and measurement characteristics

The harmonised data contained 806,097 participant-wave records, including validated terminal death rows, from 236,088 people appearing in at least one included wave; 172,518 had at least two included waves. Sample construction and denominator reconciliation are detailed in Supplementary Table S18. There were 523,570 constructed intervals comprising consecutive scheduled-wave pairs or validated terminal-death intervals across CHARLS (70,067), ELSA (67,471), HRS (180,628), KLoSA (29,954), MHAS (53,922) and SHARE (121,528) (Table 1). Median interval length was 2.0 years in five cohorts and 3.0 years in MHAS. Of 4,263 intervals shorter than 0.5 years and 12,432 longer than 5 years, 7,187 ended in death; 9,506 of the 9,508 living-endpoint extremes were MHAS intervals spanning its long scheduled wave gap. Restricting living-domain intervals to 0.5–5 years reduced the primary paired analysis by two intervals (361,562 to 361,560) and the primary transition analysis by two (266,127 to 266,125), with estimates unchanged to the displayed precision (Supplementary Tables S7 and S16).

**Table 1:** Cohort and measurement summary.

Cohort	Waves	*n* ≥ 1	*n* ≥ 2	Intervals	Cognition	Function	Death
CHARLS	1–5	25,873	23,474	70,067	4 tests	11 items	Yes
ELSA	1–9	19,802	15,388	67,471	2 tests	13 items	No^*^
HRS	5–15	36,529	33,000	180,628	27-point total	11 items	Yes
KLoSA	3–7	9,216	8,516	29,954	30-point K-MMSE	17 help items	Yes
MHAS	1–5	26,837	19,797	53,922	Memory + visual scanning/attention	10 items	Yes
SHARE	4–8	117,831	72,343	121,528	3 domains	12 items	Yes

*n* ≥ 1 counts people appearing in an included wave; *n* ≥ 2 counts people with at least two included waves. Intervals are positive-time consecutive scheduled-wave pairs or validated terminal-death intervals before model-specific complete-case restrictions. Cognition and function use non-overlapping inputs. KLoSA function records help dependence. ^*^ELSA mortality follow-up was incomplete for the analytical period and was not treated as a valid death endpoint.

The harmonisation used non-overlapping totals or component items. Both KLoSA and MHAS contained multi-item cognition. KLoSA wave-5 cognition was reconstructed from the original wave files, yielding 7,551 complete 15-item K-MMSE records at wave 5. KLoSA cognition alpha ranged from 0.822 to 0.844 and omega from 0.902 to 0.914 across waves 3–7. At the reference wave, cognition omega was 0.786 in CHARLS, 0.748 in HRS and 0.812 in SHARE. The HRS component scores reproduced cog27 exactly in all 17,516 complete records (component-score alpha = 0.496). The two-component ELSA cognition score had Pearson *r* = 0.248 and Spearman-Brown reliability = 0.397; the two-domain MHAS cognition score had *r* = 0.436 and Spearman-Brown reliability = 0.608. ADL-IADL domain correlations ranged from 0.658 to 0.819 and their Spearman-Brown coefficients from 0.794 to 0.901. These results reinforce the need to retain cohort-specific uncertainty rather than imply equal measurement precision (Supplementary Tables S1, S6 and S18).

### Continuous cognitive and functional change

**Figure 1:**
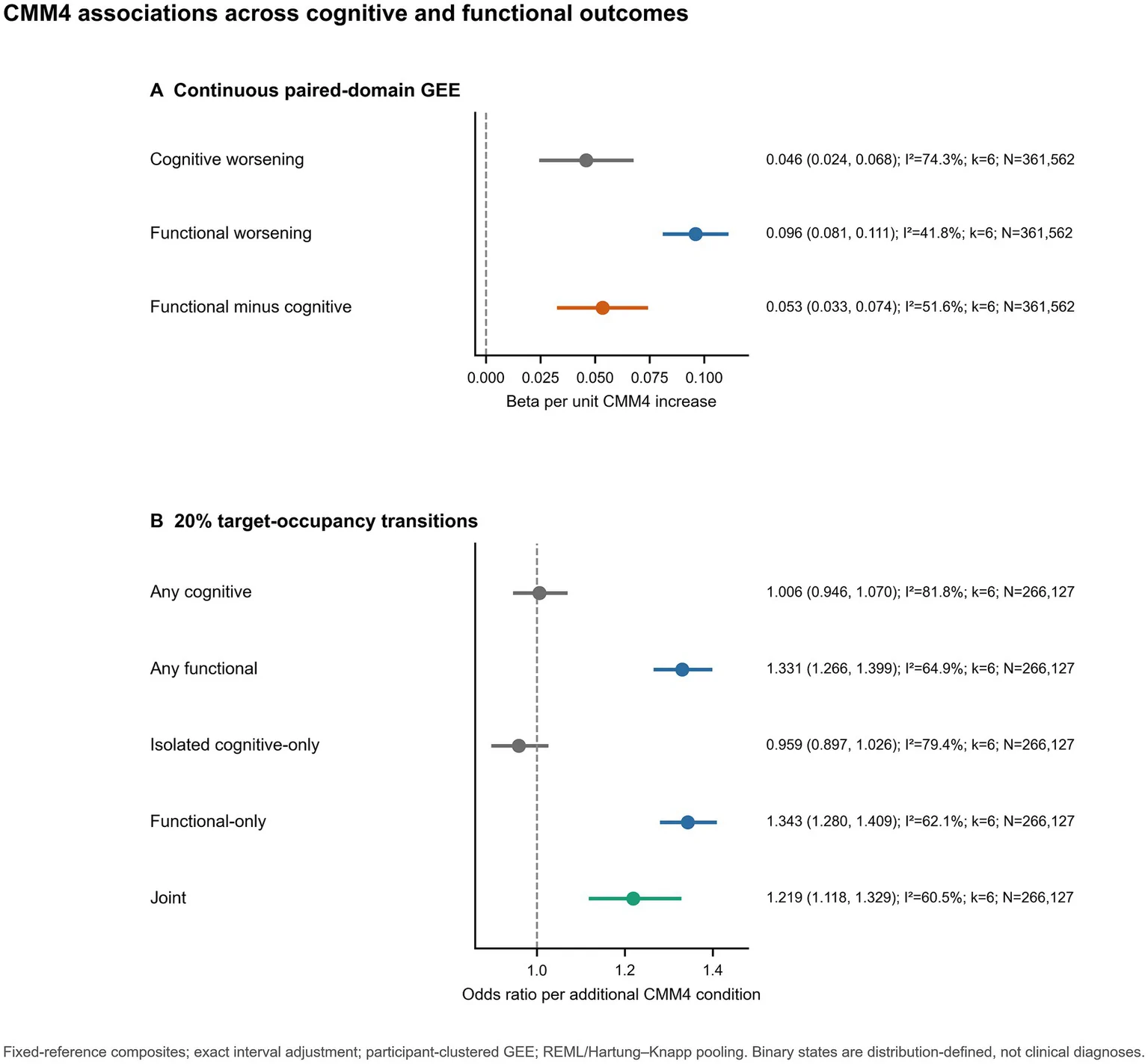
Primary pooled associations of CMM4 with cognitive and functional outcomes. Panel **(A)** shows paired-domain Gaussian GEE coefficients for cognitive worsening, functional worsening, and their formal difference. The pooled functional-minus-cognitive interaction is estimated within cohorts and therefore need not equal the arithmetic difference between the two separately pooled domain coefficients. Panel **(B)** shows logistic GEE odds ratios for any cognitive, any functional, isolated cognitive-only, functional-only, and joint transitions under cohort-wave-specific 20% target-occupancy thresholds. Estimates are pooled by REML with Hartung-Knapp confidence intervals. *n* denotes analytic intervals; *k* denotes contributing cohorts. States are distribution-defined and are not clinical diagnoses.

The paired-domain analysis included 361,562 intervals from all six cohorts. Each additional CMM4 condition accumulated was associated with cognitive worsening (*β* = 0.0459, 95% CI 0.0243 to 0.0675; prediction interval −0.0059 to 0.0976; *i*^2^ = 74.3%) and functional worsening (*β* = 0.0960, 0.0809 to 0.1111; prediction interval 0.0704 to 0.1216; *i*^2^ = 41.8%) (Table 2; Figure 1A). The formal functional-minus-cognitive contrast was 0.0534 (0.0325 to 0.0742; prediction interval 0.0084 to 0.0983; *i*^2^ = 51.6%). Thus, cognition was not unaffected, although the functional coefficient was larger.

**Table 2:** Principal results.

Outcome/analysis	k	Participant clusters	Intervals	Estimate (95% CI)	*i* ^2^
Paired cognitive worsening	6	130,672	361,562	*β* 0.0459 (0.0243–0.0675)	74.3%
Paired functional worsening	6	130,672	361,562	*β* 0.0960 (0.0809–0.1111)	41.8%
Functional minus cognitive	6	130,672	361,562	*β* 0.0534 (0.0325–0.0742)	51.6%
Any cognitive transition	6	107,207	266,127	OR 1.01 (0.95–1.07)	81.8%
Any functional transition	6	107,207	266,127	OR 1.33 (1.27–1.40)	64.9%
Functional/cognitive ratio of ORs	6	107,207	266,127	1.32 (1.23–1.42)	76.0%

Primary continuous estimates use fixed-reference paired-domain GEE. Primary binary estimates use 20% target-occupancy states. Participant clusters and analytic intervals are reported separately. Principal models include available mental-health covariates. Random-effects pooling uses REML and Hartung-Knapp confidence intervals. Secondary and sensitivity analyses are reported in Supplementary Tables S9, S13 and S16.

The approximate reliability correction increased the pooled cognitive coefficient from 0.0459 to 0.0546 (95% CI 0.0325 to 0.0767; prediction interval 0.0023 to 0.1068) and the functional coefficient from 0.0960 to 0.1062 (0.0890 to 0.1235; prediction interval 0.0751 to 0.1374). The reliability-adjusted functional coefficient remained 1.95 times the cognitive coefficient. In the cognitive-threshold simulation, measurement error attenuated median log odds most in ELSA (42% attenuation; reliability 0.397) and MHAS (36%; reliability 0.608), whereas 78–91% of the latent log odds were retained in the other cohorts. Thus, unequal reliability can weaken and destabilise thresholded cognitive associations, but it did not remove the larger functional association in continuous analyses and the simulation did not reproduce a common inverse cognitive effect (Supplementary Table S19; Supplementary Figure S2).

The separate unpaired models preserved the direction of the paired findings but yielded a substantially larger functional coefficient. After mental-health adjustment, the pooled coefficients were 0.0496 (0.0307 to 0.0684) for cognition and 0.1603 (0.1484 to 0.1721) for function. Requiring sustained confirmation of new diagnoses yielded *β* = 0.0373 (−0.0078 to 0.0823) for cognition and *β* = 0.1833 (0.1218 to 0.2447) for function. In paired models, the functional-minus-cognitive contrast remained positive for diabetes plus stroke (*β* = 0.0879, 0.0588 to 0.1170), for CMM3 excluding stroke (*β* = 0.0335, 0.0046 to 0.0623), after excluding ELSA (*β* = 0.0504, 0.0261 to 0.0746), after excluding KLoSA (*β* = 0.0560, 0.0298 to 0.0821), and with KLoSA ADL-only scoring (*β* = 0.0526, 0.0303 to 0.0749). Results across wave-relative standardisation and interval-length analyses are shown in Supplementary Tables S13 and S16 and Supplementary Figure S1.

### Primary 20% target-occupancy transitions

**Figure 2:**
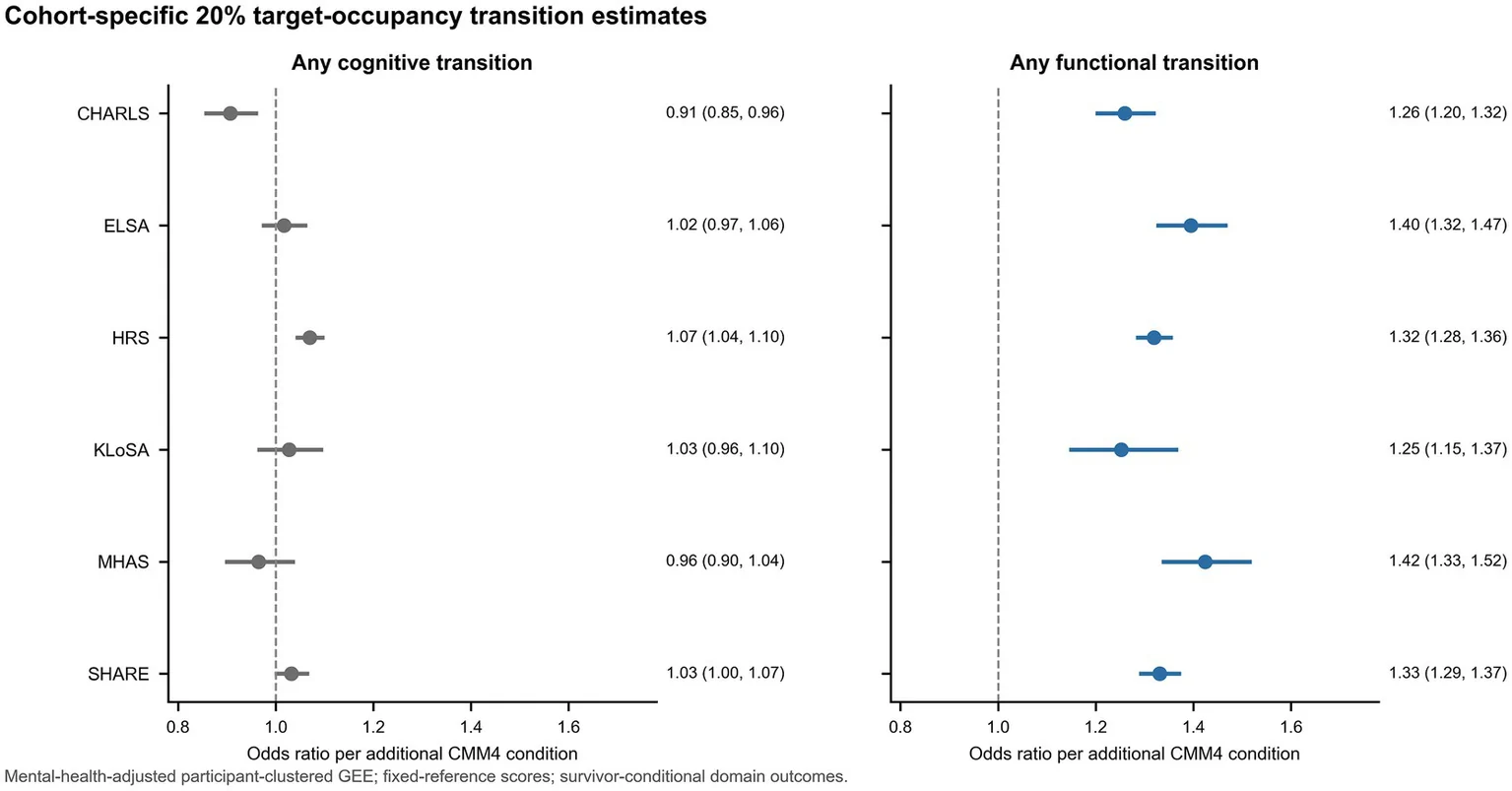
Cohort-specific CMM4 associations with any cognitive and any functional transition under the primary 20% target-occupancy definition. Models adjust for baseline cognition, baseline function, age, sex, education, exact interval length and available mental-health covariates and cluster by participant. The dashed line is *OR* = 1.

The primary transition analysis included 266,127 intervals beginning in the distribution-defined unimpaired state across all six cohorts. Each additional baseline CMM4 condition was associated with any functional transition (OR = 1.33, 95% CI 1.27 to 1.40; prediction interval 1.19 to 1.49; *i*^2^ = 64.9%) but not with a common any-cognitive transition effect (OR = 1.01, 0.95 to 1.07; prediction interval 0.86 to 1.18; *i*^2^ = 81.8%) (Table 2; Figures 1B and 2). The formal functional-to-cognitive ratio of odds ratios was 1.32 (1.23 to 1.42; *i*^2^ = 76.0%).

In the four-state decomposition, the pooled ORs were 0.96 (0.90 to 1.03; *i*^2^ = 79.4%) for isolated cognitive-only, 1.34 (1.28 to 1.41; *i*^2^ = 62.1%) for functional-only and 1.22 (1.12 to 1.33; *i*^2^ = 60.5%) for joint transition. Cohort-specific inclusive outcomes are displayed in Figure 2, and cohort-specific isolated cognitive-only estimates are reported in Supplementary Table S3. For any-cognitive transition, the direction ranged from inverse in CHARLS (OR = 0.91, 95% CI 0.85 to 0.96) to positive in HRS (OR = 1.07, 1.04 to 1.10). This substantial cognitive heterogeneity means that the near-null pooled estimate represents a mixture of opposing cohort-specific associations, not evidence that CMM is unrelated to cognitive change.

### Threshold structure, persistence and alternative definitions

**Figure 3:**
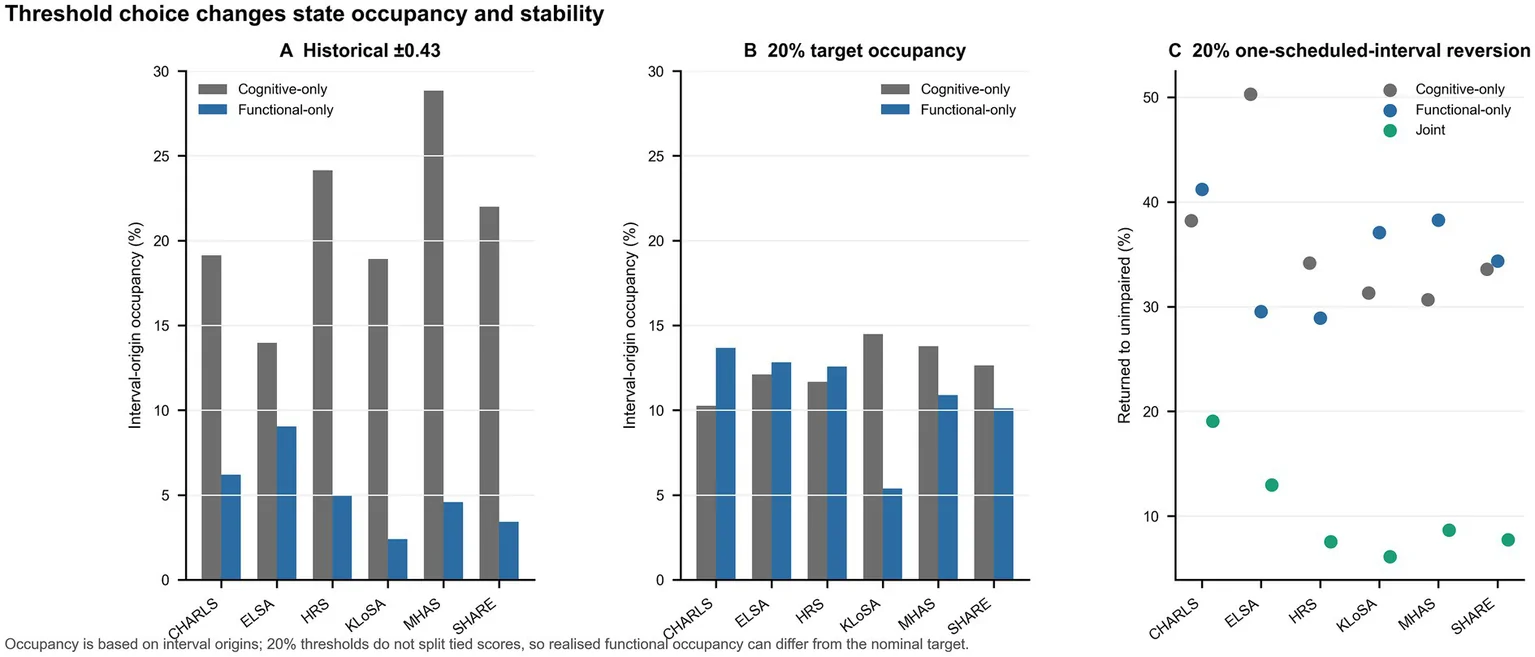
Panels **(A,B)** compare interval-origin state occupancy under the historical ±0.43 definition and the primary 20% target-occupancy definition. Panel **(C)** shows return to the unimpaired state after one scheduled interval under the primary definition. Panels **(A,B)** use the same vertical scale. Because tied functional values were not split, achieved tail occupancy can differ from 20%. Joint states were less likely than isolated states to return to unimpaired.

The 20% rule achieved a mean cognitive-tail occupancy of 19.85% across cohort-waves (range 18.30–21.47%) and a mean functional-tail occupancy of 18.06% (10.48–22.70%). The wider functional range resulted from tied floor-heavy ADL/IADL scores that were not split. At interval origins, isolated cognitive-only occupied 10.3–14.5% and functional-only 5.4–13.7% across cohorts, substantially reducing the asymmetry seen under ±0.43. Reversion to unimpaired after one scheduled interval ranged from 30.7 to 50.3% for cognitive-only, 28.9 to 41.2% for functional-only and 6.1 to 19.1% for joint states (Figure 3; Supplementary Tables S4–S5).

Persistent impairment was less common but supported the functional association. Across 173,655 eligible intervals, the persistent any-functional OR was 1.39 (1.33 to 1.45; *i*^2^ = 0%) and persistent any-cognitive OR was 0.99 (0.89 to 1.10; *i*^2^ = 77.0%). Confirmation eligibility, skipped waves, missing confirmation and deaths before confirmation are reported by cohort.

The historical ±0.43 thresholds did not select equivalent tails: across cohort-waves the mean achieved tail was 28.01% for cognition (range 16.78–44.23%) and 11.64% for function (6.97–17.16%). Under this historical definition, the mental-health-adjusted ORs were 1.04 (1.01 to 1.08; *i*^2^ = 61.4%) for any cognitive and 1.40 (1.33 to 1.47; *i*^2^ = 49.3%) for any functional transition. At −0.67/+0.67, the corresponding ORs were 1.03 (0.97 to 1.09; *i*^2^ = 84.1%) and 1.37 (1.32 to 1.43; *i*^2^ = 31.0%). Targeting 15 and 25%, respectively, yielded any-cognitive ORs of 1.03 (0.97 to 1.10) and 1.02 (0.97 to 1.08), and any-functional ORs of 1.33 (1.27 to 1.40) and 1.35 (1.27 to 1.42). Applying each cohort’s reference-wave 20% cut-points unchanged to later waves yielded OR = 1.01 (0.95 to 1.07; *i*^2^ = 81.3%) for any cognitive and OR = 1.34 (1.31 to 1.38; *i*^2^ = 25.8%) for any functional transition; across living common-domain observations, occupancy averaged 18.98% for cognition and 18.35% for function and was allowed to vary over time (Supplementary Tables S16–S17). Wave-relative standardisation at the wave-specific 20% threshold yielded OR = 1.00 (0.94 to 1.07) for cognition and OR = 1.33 (1.27 to 1.40) for function. These results show that the qualitative domain contrast was not created solely by the historical cut-points or by re-estimating cut-points every wave, while its magnitude and heterogeneity remained definition-dependent.

### Mortality, attrition and adjustment

Terminal death rows numbered 2,829 in CHARLS, 13,225 in HRS, 1,676 in KLoSA, 5,415 in MHAS and 13,584 in SHARE. Across these five cohorts, baseline CMM4 was associated with death from the unimpaired origin (OR = 1.25, 95% CI 1.08 to 1.45; *i*^2^ = 89.5%). Restricting terminal-death intervals to 0.5–5 years yielded a similar estimate (OR = 1.27, 1.10 to 1.46; *i*^2^ = 84.1%; 236,577 intervals and 99,281 participant clusters). In the secondary five-state multinomial model, only CHARLS, HRS and MHAS met convergence requirements; the pooled relative risk ratios were 1.37 (1.05 to 1.80) for death, 1.34 (1.16 to 1.54) for functional-only, 1.26 (0.93 to 1.73) for joint and 0.98 (0.77 to 1.26) for cognitive-only transition. KLoSA and SHARE multinomial models did not converge and were not pooled (Supplementary Tables S9 and S16).

The non-death response rate ranged from 55.8% in SHARE to 96.0% in KLoSA. IPCW results were nearly identical to the primary observed-living-endpoint estimates: OR = 1.006 (0.947 to 1.069) for any cognitive and OR = 1.332 (1.267 to 1.399) for any functional transition. Estimates attenuated across progressively restricted and adjusted samples. Within the held-constant CHARLS/KLoSA/SHARE cohort set, the any-functional OR changed from 1.33 in the base model (116,793 intervals) to 1.29 after mental-health adjustment (116,683), 1.28 after lifestyle adjustment (86,213) and 1.25 after socioeconomic adjustment (84,684). Comparable any-cognitive ORs were 1.00, 0.99, 1.04 and 1.03. Because covariate completeness changed the analytic sample, this sequence does not isolate covariate adjustment from complete-case restriction. Complete CMM5 analysis in four cohorts yielded OR = 0.98 (0.88 to 1.09) for any cognitive and OR = 1.23 (1.19 to 1.27; *i*^2^ = 18.3%) for any functional transition.

The temporal-boundary analysis included 62,417 participants from five cohorts. Early CMM4 accumulation was associated with later cognitive worsening (*β* = 0.0313, 95% CI 0.0152 to 0.0475) and the mean cognitive-functional outcome (*β* = 0.0237, 0.0105 to 0.0369), whereas the later functional estimate was smaller and imprecise (*β* = 0.0160, −0.0025 to 0.0345). Exposure-specific transition analyses showed a detectable any-cognitive association for diabetes plus stroke (OR = 1.10, 1.02 to 1.18), whereas CMM3 excluding stroke was null (OR = 1.00, 0.94 to 1.06). The corresponding any-functional associations remained positive for both diabetes plus stroke (OR = 1.54, 1.48 to 1.61) and CMM3 excluding stroke (OR = 1.32, 1.24 to 1.40). The cognitive transition signal was therefore concentrated in the stroke-containing exposure definition (Supplementary Table S16). These analyses reduce but cannot eliminate reverse diagnostic ascertainment, co-progression and time-varying confounding.

### Absolute risk and prediction performance

Standardised risk differences comparing CMM4 ≥ 3 with CMM4 = 0 ranged from −1.5 to 2.1 percentage points for any cognitive transition and from 3.8 to 12.6 percentage points for any functional transition across cohorts. Adding CMM4 to the base prediction model changed cross-validated AUC by −0.0002 to 0.0030 for cognition and by 0.0048 to 0.0251 for function. With baseline cognition added to the expanded base model, the corresponding changes were −0.0002 to 0.0006 and 0.0046 to 0.0222. Brier-score changes were small and calibration slopes remained close to one (Supplementary Tables S11–S12). CMM4 therefore added limited discrimination and does not constitute a screening rule.

### Model completion

Of 438 registered cohort-model cells, 376 were estimated. 62 were unavailable or failed and are listed individually in Supplementary Table S14. Most reflected documented absence of a harmonisable covariate, CMM5 unavailability in KLoSA/MHAS, ELSA mortality unavailability or insufficient events within interval strata. The KLoSA and SHARE multinomial models failed convergence. No unregistered result was pooled, and no registered cell was silently omitted.

## Discussion

CMM accumulation was associated with worsening in both continuous domains, and the formally tested functional coefficient was larger. In binary analyses using target-occupancy thresholds, CMM was associated with any functional transition across cohorts, whereas any-cognitive transition effects remained small on average and heterogeneous. Persistent functional impairment produced the most homogeneous binary result. The appropriate conclusion is therefore not that cognition is unaffected, but that functional vulnerability is more consistently associated with CMM while harmonised binary cognitive transitions do not share a stable cross-cohort mean association.

Inclusive domain outcomes avoided depletion of cognitive events through assignment to the joint state. Target-occupancy thresholds also reduced the severity asymmetry produced by applying identical *z* cut-points to a relatively symmetric cognitive distribution and a floor-heavy functional distribution, while fixed-reference standardisation retained change relative to a stable cohort benchmark. The item-level audit confirmed multi-item cognition in KLoSA and MHAS, although reliability and construct breadth still differed between cohorts.

The smaller continuous cognitive association is consistent with longitudinal reports linking cardiometabolic multimorbidity to cognitive decline and dementia risk, although previous studies differ in disease definitions, cognitive batteries and follow-up horizons (6, 7, 9–12). A continuous score can detect small, widely distributed shifts that do not move many participants across a binary threshold. Threshold crossing is additionally sensitive to baseline distance from the cut-point, tied scores and cohort-specific measurement precision. The inverse any-cognitive association in CHARLS and positive association in HRS persisted across several state definitions, arguing against a single threshold choice as the sole explanation. However, this contrast cannot be assigned confidently to true epidemiologic effect modification because the cohorts also differ in cognitive content, education and language, cardiometabolic ascertainment, baseline risk and selective observation. The reliability correction and simulation showed that measurement precision can materially attenuate thresholded cognitive associations, especially in ELSA and MHAS, but did not by itself explain the CHARLS-HRS directional contrast. These distinctions help explain why delayed continuous cognitive worsening was detectable while the pooled any-cognitive transition estimate remained near one and highly heterogeneous.

The more consistent functional association accords with evidence that multimorbidity predicts physical-function decline and disability (8, 14). Zhang et al. (14) examined longer-term joint trajectories of disability, depression and cognition, whereas the present analysis compares paired adjacent-wave changes and inclusive cognitive and functional transitions. Functional thresholds identified a floor-heavy upper tail of ADL/IADL limitation, which may capture a more advanced or persistent state than low cognitive performance; the matched-occupancy and persistent analyses reduce, but do not eliminate, that construct difference. The functional contrast should therefore be interpreted as a cross-cohort pattern under the measured outcomes rather than proof of a domain-specific biological mechanism.

The remaining domain contrast is supported by several complementary findings: the paired continuous interaction excluded zero; the primary binary ratio of odds ratios exceeded one; persistent functional impairment was associated with CMM with no detected between-cohort heterogeneity; and IPCW estimates were nearly unchanged. The continuous cognitive association was less robust than the functional association: under the sustained-diagnosis sensitivity, the cognitive estimate attenuated and its confidence interval crossed zero, whereas the functional estimate remained positive. The substantially larger functional coefficient in unpaired analyses may partly reflect outcome-specific sample composition, because intervals with observed function but missing cognition entered the unpaired functional model but not the paired comparison. The paired model was retained as primary because it compares both domains within the same observed intervals; its smaller functional coefficient is therefore more conservative in magnitude. However, the cognitive prediction interval included effects in both directions, the functional binary estimate retained moderate heterogeneity, and sequential covariate adjustment attenuated the association. These observations argue against a universal biological function-specific pathway.

The strongest and most consistent results were synchronous, but the temporal-boundary analysis also detected a smaller delayed cognitive and mean cognitive-functional association; delayed functional change was not statistically distinguishable from zero. One plausible interpretation is that cumulative vascular, metabolic and cerebrovascular injury may emerge gradually in cognition, whereas measured ADL/IADL limitation is more closely coupled to concurrent disease burden, acute health changes and recovery. The contrast should remain cautious: the delayed cognitive and functional coefficients were not compared by a formal interaction test, and the imprecise functional estimate does not prove the absence of a delayed functional pathway. The restriction of the binary cognitive signal to the diabetes-plus-stroke count is consistent with a contribution from cerebrovascular injury, but may also reflect stroke’s more discrete clinical ascertainment and larger immediate effect on test performance. A newly recorded diagnosis may nevertheless follow increased healthcare contact caused by declining health. Carry-forward, sustained-diagnosis, diabetes-plus-stroke and CMM3-without-stroke sensitivities reduce specific ascertainment concerns but cannot eliminate reverse detection, unmeasured disease severity, the mortality burden associated with CMM, or time-varying confounding (28). The results should not be interpreted causally.

Strengths include six large ageing cohorts, exact calendar time, a common row-complete CMM4 definition, non-overlapping composites, participant-clustered models, formal paired-domain tests, matched and persistent states, fixed reference-wave thresholds, disease-restricted exposures, mortality endpoints, IPCW, held-constant-cohort adjustment, explicit reliability correction and simulation, and a complete model-failure matrix. Limitations include heterogeneous cognitive batteries, limited breadth and low Spearman-Brown reliability for the two-component ELSA cognitive composite, reliance on classical-error assumptions in the reliability sensitivity, self-reported diagnoses, incomplete ELSA mortality follow-up, non-convergence of two multinomial models, complete-case domain scoring, residual confounding and limited added discrimination. Mortality and selective attrition can alter the composition of observed longitudinal samples despite explicit competing-death modelling and IPCW (29, 30). Two-component constructs were assessed with correlation and Spearman-Brown coefficients rather than omega. The matched thresholds remain distribution-defined, and tied floor scores prevented exact target occupancy in several functional waves.

Clinically, CMM may be a prompt to consider functional status as one part of comprehensive assessment consistent with integrated-care approaches to intrinsic capacity (13), but the observed risk differences and small incremental AUC do not support a stand-alone triage or screening rule. The binary states do not diagnose mild cognitive impairment, dementia or dependence, and no number-needed-to-screen is claimed.

## Conclusion

CMM was associated with worsening in both cognition and function, with a larger functional coefficient, and with more consistent functional than cognitive transitions across cohorts. Reliability correction modestly increased both continuous coefficients without removing the functional contrast, while simulation showed that lower cognitive reliability can attenuate thresholded associations. The functional pattern remained directionally similar under fixed reference-wave thresholds, persistent states, disease-restricted exposures and mortality-valid analyses, while IPCW estimates for non-death attrition were nearly unchanged. Cognitive transition estimates varied from inverse in CHARLS to positive in HRS and were concentrated in the stroke-containing restricted exposure, underscoring that effect size and direction depend on cohort, measurement and outcome definition. The results describe cross-cohort cognitive-functional vulnerability rather than a causal pathway or validated screening rule.

## Data Availability

Participant-level cohort data remain subject to the access conditions of HRS, ELSA, CHARLS, KLoSA, MHAS and SHARE and cannot be redistributed with this article. The harmonisation and analysis scripts, configuration files, aggregate outputs, model-completion records, figures, validation materials and software requirements are publicly available in a data-free reproducibility package at GitHub: https://github.com/mccrumbschorsch-spec/cmm-cognitive-functional-transitions. The version corresponding to this revision (v1.0.1) is permanently archived in Zenodo: https://doi.org/10.5281/zenodo.22055237.

## Supplementary material

SUPPLEMENTARY FIGURE S1 Key sensitivity analyses for any-cognitive and any-functional transitions. Estimates show odds ratios per additional condition for the primary CMM4 analysis, fixed reference-wave 20% thresholds, diabetes-plus-stroke exposure, CMM3 excluding stroke, exclusion of ELSA, exclusion of KLoSA and KLoSA ADL-only functional scoring. Disease counts differ in range, so restricted-exposure estimates should be interpreted within rather than directly between exposure definitions.

SUPPLEMENTARY FIGURE S2 Reliability-based sensitivity analyses. Panel **(A)** compares observed pooled continuous coefficients with approximate reliability-adjusted coefficients; bars are 95% Hartung-Knapp confidence intervals, and prediction intervals are reported separately in Supplementary Table S19. Panel **(B)** shows cohort-specific latent and after-measurement-error odds ratios from 1,000 cognitive-threshold simulation repetitions; points are medians and bars are 2.5th-97.5th simulation percentiles. Reliabilities are reference-wave omega or Spearman-Brown coefficients, as appropriate. Panel **(B)** is an illustrative thresholded analogue of the continuous CMM-accumulation estimand and is not a correction of the primary baseline-CMM4 transition OR.
