# Supplementary material for: Cardiometabolic multimorbidity and cognitive-functional transitions across international ageing cohorts
Source: Front Public Health. 2026 Sep 10;14:1920385. doi: 10.3389/fpubh.2026.1920385 (PMC13600987; doi:10.3389/fpubh.2026.1920385)
Supplement: Supplementary file 1 [file Supplementary_file_1.DOCX]

Supplementary Appendix 1: Reconstruction, harmonisation and statistical methods

# 1. Reconstruction scope

The revision was rerun from the six cohort participant-wave files rather than by manipulating archived model summaries. Upstream Stata processing files and release documentation were reviewed against the exported analysis files. The final pipeline writes only cohort-level and pooled aggregate outputs. Participant-level intermediate files remain outside the reproducibility package.

The core-model analysis lock is dated 21 August 2026; the reviewer-requested reliability sensitivity was locked on 23 August 2026. The configuration identifies every cohort release, wave sequence, time field, cognitive and functional input, CMM component, missing-value rule, covariate, mortality endpoint and analysis option. A SHA-256 manifest records input and script hashes.

# 2. Cohort provenance and waves

CHARLS used Harmonized CHARLS Version D (June 2021), locally extended with the 2020 survey, waves 1-5. ELSA used Harmonized ELSA Version G.3 (2002-2019), waves 1-9. HRS used Harmonized HRS Version D (1992-2021) with RAND HRS 1992-2020 Version 2, analytical waves 5-15. KLoSA used Harmonized KLoSA Version E.2 (2006-2020), July 2023, DOI 10.34729/815E-WN35; cognition was reconstructed for waves 3-7. MHAS used Harmonized MHAS Version C.2 (2001-2018), August 2023, waves 1-5. SHARE used Harmonized SHARE Version F.2 (2004-2020), April 2024, based on SHARE Release 9.0.0; the common cognitive-functional analysis used waves 4-8.

The exported CSV files were checked against their cohort working DTA files. Row and column counts matched, identifier-wave pairs were unique, and representative values were reconciled. A reproducible extraction script now documents the final DTA/CSV and KLoSA cognition steps that were absent from the archived package.

# 3. Exact time and scheduled intervals

Interview year and month were used to compute decimal calendar year as year+(month−0.5)/12. Months outside 1-12 were rejected. Cohort wave sequences were declared before the data were read. Scheduled adjacent intervals required consecutive entries in that sequence, a positive calendar duration and the variables required for the model. A participant observed at waves 1 and 3 but not wave 2 did not contribute a scheduled wave-1-to-wave-3 interval.

The exact interval audit is in Supplementary Table S7. Median interval duration was 2.0 years in CHARLS, ELSA, HRS, KLoSA and SHARE and 3.0 years in MHAS. The interquartile ranges were 2.00-2.92, 1.92-2.17, 1.75-2.17, 1.92-2.00, 2.08-3.17 and 1.83-2.25 years, respectively. Full ranges are retained because some interviews were early, late or followed longer calendar gaps even within a scheduled wave pair.

# 4. CMM construction

## 4.1 Complete CMM4

The common primary count contained hypertension, diabetes, heart disease and stroke. Each component was recoded through an explicit positive, negative and missing-code schema. Undeclared non-numeric or out-of-range values stopped the pipeline. For self-reported ever-diagnosed conditions, a first positive report was carried forward across later living interviews, including overriding a later observed zero. Terminal death rows were not used to score interview-derived CMM.

CMM4 required all four components within a participant-wave. Interval exposure change required complete CMM4 at both endpoints. This prevents a condition from appearing or disappearing because the number of measured components changed.

## 4.2 CMM5, sustained diagnoses and restricted-condition counts

The CMM5 sensitivity added cholesterol in CHARLS, ELSA, HRS and SHARE and required all five components at both endpoints. KLoSA and MHAS were not assigned a pseudo-CMM5. The sustained-diagnosis sensitivity required a newly positive condition to be positive at the next scheduled living interview. A missing confirmation, skipped scheduled wave or death before confirmation did not count as confirmation.

Two additional counts addressed ascertainment concerns directly. The diabetes-plus-stroke count retained two conditions with comparatively discrete clinical ascertainment, and the CMM3 count excluded stroke while retaining hypertension, diabetes and heart disease. Each restricted count required the same complete component set at both interval endpoints. These analyses supplement rather than replace the sustained-diagnosis analysis.

CHARLS 2018 stroke was reconstructed from the original 2018 health-status file because the archived merge had omitted the wave. This was a source-processing correction, not an imputation.

# 5. Cognitive composites

The central rule was one validated total or a fixed set of non-overlapping components, never both. Component scores were converted to the same direction, standardised by the first eligible reference-wave mean and standard deviation, and averaged with declared domain weighting. All specified components were required.

## 5.1 CHARLS

Reference wave: 1. Inputs: total immediate plus delayed 10-word recall (tr20, 0-20), orientation (orient, 0-4), serial subtraction (ser7, 0-5), and figure copy (draw, 0-1). Immediate recall, delayed recall, recall means, cognition totals and standardised totals were excluded because they overlap these inputs.

## 5.2 ELSA

Reference wave: 1. Inputs: total immediate plus delayed recall (tr20, 0-20) and orientation (orient, 0-4). Numeracy was not used in the common composite because it was absent or sparsely administered in several waves; verbal fluency was not available at wave 6. The two-component breadth limitation is acknowledged.

## 5.3 HRS

Reference wave: 5. The validated 27-point cognition total (cog27, 0-27) was used alone in the analytical composite. Its recall, backward-count/working-memory and serial-subtraction components were not added to the total. For the reliability audit only, the non-overlapping component scores tr20, bwc20 and ser7 were extracted at wave 5; their sum matched cog27 in all 17,516 complete records. The archived component exports were incomplete at waves 14-15, while cog27 retained broad coverage.

## 5.4 KLoSA

Reference wave: 3. The 30-point K-MMSE was rebuilt from 15 non-overlapping scored items: weekday, date, season, place, address, immediate recall, five serial subtractions, delayed recall, two naming items, sentence repetition, three-step command, reading/obeying, writing and figure copy. Total recall, the archived cognition total and dementia group were excluded to avoid overlap.

The archived wave-5 merge loaded the new-sample imputation file and merged the old-sample file without updating overlapping cognition fields; only 888 wave-5 records retained cognition. The reconstruction extracted the first imputation from the source wave files with dedicated names and yielded 7,551 complete wave-5 records. The archived season and reading-task variables also had collision/intermediate-coding problems and were reconstructed from source fields. Wave 8 lacked a same-structure cognition imputation file and was excluded from the common cognitive analysis.

## 5.5 MHAS

Reference wave: 1. Immediate and delayed eight-word recall were standardised and averaged into a memory domain; that domain was then averaged with standardised visual scanning/attention (vscan, 0-60). The total recall score was excluded as an overlapping sum. Drawing scales changed range between waves, verbal fluency had changing observed maxima, serial subtraction was available only late, and orientation did not span all waves; these were not mixed into the common composite.

## 5.6 SHARE

Reference wave: 4. Immediate and delayed 10-word recall were averaged as a memory domain, then combined equally with serial subtraction (ser7, 0-5) and verbal fluency (verbf, 0-100). Total recall was excluded as an overlapping sum. Orientation and numeracy were not included because of selective/sparse administration.

# 6. Functional composites

Function was harmonised as greater limitation. Each cohort first formed the mean/fraction of a fixed ADL item set and a fixed IADL item set; the two domains were reference-standardised and equally averaged. All items within each domain were required in the primary construction.

CHARLS used six ADLs and five IADLs (11 items). ELSA used six ADLs and seven IADLs (13). HRS used six ADLs and five IADLs (11). KLoSA used seven ADLs and ten IADLs (17). MHAS used six ADLs and four IADLs (10). SHARE used six ADLs and six IADLs (12). Totals and dependency variables were excluded when their items were used.

KLoSA differs conceptually: its indicators record need for help, not difficulty. The 17 items were dressing, bathing, eating, toileting, transferring, brushing and continence; meal preparation, shopping, medication use, money management, telephone use, transportation, going out, laundry, housework and grooming. This construct is identified explicitly in all tables.

A KLoSA ADL-only sensitivity retained the seven help-dependence ADLs and omitted all ten IADLs. A separate exclusion sensitivity removed KLoSA entirely. These analyses test whether the pooled functional contrast depends on mixing KLoSA help-dependence with difficulty-based measures in the other cohorts.

# 7. Reliability and distribution audits

Complete-case coefficient alpha and one-factor omega were computed at each wave only for constructs with at least three scored components. Two-component constructs were summarised by their Pearson inter-component correlation and Spearman-Brown coefficient; omega was not reported because a two-indicator one-factor model does not provide a stable, uniquely identified reliability estimate without additional constraints. Reliability is descriptive because heterogeneous batteries do not imply measurement invariance.

At the reference wave, cognition alpha/omega were 0.635/0.786 in CHARLS, 0.496/0.748 in HRS and 0.651/0.812 in SHARE. The HRS component scores exactly reproduced cog27 in all 17,516 complete records; its omega is a Pearson one-factor coefficient across three component scores, not item-level ordinal omega. KLoSA item-level alpha/omega across waves 3-7 were 0.822-0.844/0.902-0.914. ELSA's recall-orientation composite had r=0.248 and Spearman-Brown=0.397; MHAS's memory-attention composite had r=0.436 and Spearman-Brown=0.608. Because function was constructed from two top-level ADL and IADL domains, it was also summarised with correlation and Spearman-Brown rather than omega: r ranged from 0.658 to 0.819 and Spearman-Brown from 0.794 to 0.901 across cohorts.

## 7.1 Reliability-based sensitivity analyses

For the continuous outcomes, we applied an approximate correction for classical outcome measurement error. Each cohort-specific coefficient and its standard error were divided by the square root of the corresponding reference-wave reliability estimate, after which the corrected cohort estimates were pooled using the same REML variance estimator and Hartung-Knapp confidence interval. Cognition reliabilities were omega for CHARLS, HRS, KLoSA and SHARE and Spearman-Brown for ELSA and MHAS. Function reliabilities were the ADL-IADL Spearman-Brown coefficients. The correction assumes reliability is transportable from the reference wave and that measurement error is independent of CMM and covariates; it is therefore a sensitivity analysis, not a definitive errors-in-variables estimator.

For the threshold simulation, each cohort's latent baseline and endpoint cognitive scores were generated as correlated standard normals using the empirical baseline-to-end cognitive correlation from the exact paired fixed-reference mental-health-adjusted sample. CMM4 change was sampled from that cohort's empirical distribution. The cohort-specific observed continuous coefficient was divided by the square root of reliability to define the latent association. Independent Gaussian measurement error with variance (1-r)/r was then added at each occasion, after which cognitive worsening was defined by transition from above the 20th percentile at baseline to at or below the 20th percentile at follow-up. We fitted an unadjusted logistic model per simulated CMM4 increment to the latent and error-contaminated outcomes. The exercise used 1,000 repetitions per cohort, a fixed seed, and at most 30,000 intervals per repetition; all 1,000 repetitions were estimable in every cohort. It is a transparent thresholding analogue of the continuous CMM-accumulation estimand, not a replacement for the covariate-adjusted baseline-CMM4 GEE transition model.

The approximate correction changed the pooled cognitive coefficient from 0.0459 to 0.0546 (95% CI 0.0325-0.0767; prediction interval 0.0023-0.1068) and the functional coefficient from 0.0960 to 0.1062 (0.0890-0.1235; prediction interval 0.0751-0.1374). The adjusted functional coefficient remained 1.95 times the cognitive coefficient. In the simulation, the median proportion of latent log odds retained after measurement error was 0.58 in ELSA, 0.64 in MHAS and 0.78-0.91 in the other cohorts. Full inputs, percentiles and estimand boundaries are reported in Supplementary Table S19 and Supplementary Figure S2.

## 7.2 Distribution audit

For each cohort-wave-domain, the distribution audit reports N, mean, SD, skewness, minimum, maximum, quantiles, observed-minimum occupancy, observed-maximum occupancy and zero occupancy. Functional source metrics had large observed floors, typically approximately 69%-88%, explaining why an exact target tail could not always be obtained without splitting tied scores.

# 8. Standardisation and continuous outcomes

Fixed-reference standardisation was primary. Wave-relative standardisation, which forces each wave to mean zero and SD one, was retained only as sensitivity analysis. The primary continuous outcomes were unannualised cognitive and functional worsening. Exact interval years entered as a covariate rather than dividing both exposure and outcome by the same duration. Annualised, wave-relative, central-interval and interval-stratified models were secondary. An additional audit counted intervals shorter than 0.5 years and longer than 5 years, distinguished terminal-death from living endpoints, and repeated the principal models after restricting durations to 0.5-5 years.

Across all constructed intervals, 4,263 were shorter than 0.5 years and 12,432 were longer than 5 years. Of these 16,695 extremes, 7,187 ended in a validated death endpoint. Among 9,508 extremes with a living endpoint, 9,506 were MHAS intervals spanning its long scheduled gap and two were HRS interviews. These intervals contributed almost no complete principal-model observations: the duration restriction reduced the paired-domain analysis and primary transition analysis by two intervals each, and pooled estimates were unchanged to the displayed precision. The mortality sensitivity separately restricted validated terminal-death intervals to 0.5-5 years; the pooled death OR was 1.27 (95% CI 1.10-1.46; I²=84.1%) across five mortality-valid cohorts, compared with 1.25 (1.08-1.45; I²=89.5%) without the restriction. No proxy indicator entered the domain models; terminal-death rows had cognition, function and interview-derived CMM masked.

The paired-domain analysis retained only intervals with both outcomes. The stacked GEE included baseline CMM4 and domain interactions with CMM4 change, baseline cognition, baseline function, age, sex, education, interval length and adjustment variables. Cognitive was the reference. From the fitted coefficient vector and covariance matrix, the pipeline exported the cognitive slope, functional slope and functional-minus-cognitive contrast with correct uncertainty. Reported N is paired intervals, not twice the stacked-row count.

# 9. Binary states and the ±0.43 issue

## 9.1 Primary target-occupancy algorithm

For each cohort-wave, thresholds were estimated on living records with both domains observed. The algorithm considered observed values without splitting tied scores, chose the threshold with realised tail occupancy closest to 15%, 20% or 25%, and chose the stricter/lower-occupancy threshold if deviations tied. The same common-sample denominator was used for cognition, function and state construction. Target and realised occupancy, deviation, lower and upper empirical CDF bounds and tie mass were retained.

## 9.2 Historical thresholds

The archived thresholds were cognition z≤−0.43 and function z≥+0.43. Under a standard normal distribution, Φ(−0.43)=0.3336 and 1−Φ(0.43)=0.3336. This arithmetic is compatible with a one-third-tail heuristic, but no contemporaneous analysis record proves that a formal tertile rule was the original derivation. The recorded rationale was cross-cohort comparability plus adequate transition events. The revision therefore does not invent a post hoc rationale: it reports the arithmetic, the missing design record and the observed empirical consequences.

Across cohort-waves, historical thresholds selected a mean 28.01% cognitive tail (range 16.78%-44.23%) and 11.64% functional tail (6.97%-17.16%). The historical definition is retained for reproducibility only. It does not map to mild cognitive impairment, dementia or dependence.

## 9.3 Fixed reference-wave 20% thresholds

For the absolute-position sensitivity, each cohort's 20% cognitive and functional cut-points were estimated once on the living common-domain sample at its declared reference wave. Those numeric cut-points were then locked and applied unchanged to all later fixed-reference scores. Later occupancy was not forced back to 20%. Across 40 evaluable cohort-waves, mean occupancy was 18.98% for cognition (range 12.07%-30.64%) and 18.35% for function (10.48%-24.57%). The corresponding pooled transition ORs were 1.01 (95% CI 0.95-1.07) for any cognitive and 1.34 (1.31-1.38) for any functional impairment.

## 9.4 Persistent states

Persistent any-cognitive impairment required cognitive impairment at the endpoint and at the next scheduled wave; persistent any-functional used the analogous rule. The confirmation wave had to be the next declared scheduled wave and living. The audit reports eligible endpoints, missing confirmations, skipped waves, deaths before confirmation, events and elapsed confirmation time. These endpoints are confirmation/survivor-selected and are interpreted as sensitivity analyses rather than unbiased population transition probabilities.

# 10. Statistical models

Gaussian identity-link and logistic GEE used participant clusters and an independence working correlation structure. Primary binary models used the Mancl-DeRouen bias-reduced sandwich covariance as implemented by statsmodels cov_type="bias_reduced"; continuous models used the participant-clustered robust sandwich covariance. Transition models adjusted for baseline cognition, baseline function, age, sex, education, exact interval length and the declared adjustment set. Binary models required minimum event and non-event counts and finite target estimates with positive standard errors. Non-converged models never entered pooling.

The binary paired-domain model stacked any-cognitive and any-functional outcomes on the same intervals and allowed domain-specific nuisance slopes. The CMM-by-domain interaction was exponentiated as a ratio of odds ratios, not labelled an ordinary OR.

The secondary multinomial model used unimpaired as the explicit reference and required all four living destinations; mortality-valid cohorts additionally required death events. It was fitted by Newton-Raphson optimisation with a maximum of 200 iterations. A successful optimiser convergence flag, finite estimates and positive finite standard errors were required. Covariance clustered by participant. A mortality-valid cohort with no death events could not be labelled a five-state model.

Cohort estimates were pooled by REML with Hartung-Knapp confidence intervals. Prediction intervals were reported for k≥3. I² was calculated from Cochran's Q. Pooling metadata retain the contributing cohorts, covariates, mortality availability, analytic N and clusters after excluding invalid estimates.

Measurement and ascertainment sensitivities used the same modelling and pooling rules. They excluded ELSA, excluded KLoSA, replaced KLoSA function with the seven ADL help-dependence items, substituted the diabetes-plus-stroke count, or substituted CMM3 excluding stroke. The disease-restricted counts have different ranges from CMM4 and are interpreted within, not directly between, exposure definitions.

The temporal-boundary analysis was participant-level and required at least four observed waves. Early CMM4 slope was separated from later domain slope at a non-overlapping boundary. Models adjusted for CMM4, cognition and function at the boundary, age, sex, education, and durations of the early and later periods. Five cohorts contributed 62,417 participants; MHAS lacked enough compatible repeated waves. This design strengthens temporal separation but does not identify a causal effect.

# 11. Mortality and IPCW

Mortality endpoints were checked as absorbing terminal rows. Domain and interview-derived CMM scores were masked on death rows so that carried placeholders could not alter reference or wave distributions. ELSA was labelled mortality unavailable because its available mortality follow-up ended before the analytical interview follow-up. The 0.5-5-year mortality sensitivity retained only validated terminal-death intervals in that duration range and continued to adjust for exact interval length.

Non-death response models used a participant-by-scheduled-wave risk table reconstructed from cohort response histories. Deaths were removed from the censoring model. Predictors included prior response, CMM, cognition, function, mental health and sociodemographics. Stabilised weights were truncated at cohort-specific first and 99th percentiles. Response ranged from 55.85% in SHARE to 95.98% in KLoSA; weight medians were 0.962-0.994 and truncated maxima 1.09-1.62.

# 12. Absolute risk and prediction

Prediction used five-fold StratifiedGroupKFold, preserving participant groups and checking events in every fold. The base prediction model contained age, sex, education and baseline function; the expanded model additionally contained baseline cognition. Each model was evaluated with and without CMM4 on the same folds. AUC and Brier-score intervals and their changes used 200 participant bootstrap samples. Calibration intercept and slope are point estimates; unstable or separated calibration models were declared not estimable.

Standardised risks were generated for CMM4≥3 and CMM4=0 after verifying events and non-events in both exposure groups. Risks and differences are conditional on an observed living endpoint and adjust for interval length. Their reciprocal is not called a number-needed-to-screen.

# 13. Sequential adjustment and missing covariates

The base set contained age, sex, education, baseline cognition, baseline function and exact interval. Continuous change-on-change models additionally contained baseline CMM4; transition models treated baseline CMM4 as the exposure. Mental-health models added the available depression and psychiatric variables. Lifestyle models added smoking, BMI, physical activity and alcohol where available. Socioeconomic models added wealth or income and marital status where available. Polypharmacy and medication adherence were not sufficiently harmonisable. Each model records its actual covariates. Held-constant-cohort meta-analysis prevents apparent attenuation from arising solely because cohorts dropped out, but complete-case interval counts still change as additional covariates are introduced. These sequences are therefore interpreted as progressively restricted and adjusted samples rather than as a pure decomposition of confounding.

# 14. Model-completion rules

The configuration generated the expected cohort-by-analysis registry before modelling. Each cell was classified as estimated, failed, skipped because a required variable was unavailable, or ineligible because of event/variation rules. The final registry contained 438 cells: 376 estimated and 62 failed/skipped. The two principal numerical non-convergences were KLoSA and SHARE multinomial transitions. No result existed outside the registry, and no registered cell was silently absent.

# 15. Software and reproducibility boundary

The harmonisation and statistical analysis used Python 3.14.2 with pandas 2.3.3, NumPy 2.4.3, SciPy 1.17.1, statsmodels 0.14.6, patsy 1.0.2 and scikit-learn 1.8.0. KLoSA wave extraction used R 4.5.2 with haven 2.5.5. The package contains the filled configuration, input builder, KLoSA extractor, v3 analysis pipeline, binary/prediction finaliser, cohort and HRS reliability audits, reliability-calibration and measurement-error simulation scripts, primary and supplementary figure generators, validation scripts and regression tests. It excludes cohort data and all participant-level outputs. Aggregate CSVs, JSON manifests, figures and the complete failure/completion records are included.

# 16. Ethics and data governance

The six parent studies document original ethics approval and informed consent. Access routes differ: some resources are openly downloadable after registration, whereas others require data-use registration or agreement. We therefore use the accurate phrase “public, registered or controlled-access deidentified cohort data,” not unrestricted public data.

The applicable national basis is Article 32 of the Measures for Ethical Review of Life Science and Medical Research Involving Humans, National Health Commission document No. 4 of 2023, issued 18 February 2023. It permits exemption for research using lawfully obtained public data or anonymised information when the stated safeguards are met. This study used lawfully accessed anonymised information and involved no recruitment, recontact, intervention or direct identifiers; it was therefore treated as exempt from additional ethics review under Article 32. No separate local approval number is claimed. Official source: https://www.nhc.gov.cn/qjjys/c100016/202302/6b6e447b3edc4338856c9a652a85f44b.shtml.
